# Positive association between musclin and insulin resistance in obesity: evidence of a human study and an animal experiment

**DOI:** 10.1186/s12986-017-0199-x

**Published:** 2017-07-10

**Authors:** Wen-Jia Chen, Yue Liu, Yu-Bin Sui, Hong-Tao Yang, Jin-Rui Chang, Chao-Shu Tang, Yong-Fen Qi, Jing Zhang, Xin-Hua Yin

**Affiliations:** 1 Department of Cardiology, the First Affiliated Hospital of Harbin Medical University, Harbin, 150001 China; 2 School of P.E. and Sports Science, Beijing Normal University, Beijing, 100875 China; 3 Key Laboratory of Molecular Cardiovascular Science, Ministry of Education, Peking University Health Science Center, Beijing, 100191 China

**Keywords:** Musclin, Obesity, Insulin resistance, Skeletal muscle, Endoplasmic reticulum stess

## Abstract

**Background:**

Musclin is a novel skeletal muscle-derived secretory factor considered to be a potent regulator of the glucose metabolism and therefore may contribute to the pathogenesis of obesity and insulin resistance (IR).

**Methods:**

To test this hypothesis, we examined the plasma musclin levels in overweight/obese subjects and lean controls. Rats on a high fat diet (HFD) were used as the annimal model of obesity. Radioimmunoassay and western blot were used to determine musclin levels in plasma and skeletal muscle.

**Results:**

According to radioimmunoassays,the overweight/obese subjects exhibited elevated musclin plasma levels compared with the lean controls (89.49 ± 19.00 ng/L vs 80.39 ± 16.35 ng/L, *P* < 0.01). The musclin levels were positively correlated with triglyceride, fasting plasma glucose, and homeostasis model assessment of IR levels. These observations were confirmed with a high-fat diet(HFD) rat model. HFD rats also exhibited increased musclin immunoreactivity in plasma (*P* < 0.01) and in skeletal muscle (*P* < 0.05), as well as increased musclin mRNA levels in skeletal muscle (*P* < 0.01). Musclin incubation significantly inhibited muscles ^3^H-2-DG uptake in the normal diet(ND) group (*P* < 0.01). The protein expression of glucose transporter type 4 was significantly down regulated by 30% (*P* < 0.05) in the ND group after soleusmuscle was incubated with musclin compared with the control. Musclin incubation also increased the protein levels of glucose-regulated protein (GRP)78 and GRP94 by 146.8 and 54% (both *P* < 0.05), respectively, in ND rats.

**Conclusions:**

Our data support the hypothesis that musclin has a strong relationship with obesity-associated IR by impairing the glucose metabolism and, at least in part, through causing endoplasmic reticulum stress.

**Electronic supplementary material:**

The online version of this article (doi:10.1186/s12986-017-0199-x) contains supplementary material, which is available to authorized users.

## Background

Individuals with obesity are at a higher risk for obesity-associated medical conditions, such as metabolic syndrome, and insulin resistance (IR), which can develop into type 2 diabetes mellitus (T2DM) [1]. Although the precise mechanism linking obesity to IR and T2DM are unknown, extensive evidence suggests that impaired glucose disposal in skeletal muscle plays a crucial role in development of obesity-associated disease [2]. Recently, it has been shown that skeletal muscle is an endocrine organ that can regulate energy metabolism homeostasis by releasing a variety of bioactive factors [3]. Pedersen et al. reported that cytokines and peptides released by muscle fibers exert either paracrine or endocrine effects and could be classified as “myokines” [4]. To date, a number of studies have shown that myokines, such as interleukin-6 (IL-6), interleukin-15 (IL-15) and brain-derived neurotrophic factor(BDNF), may be potential regulators of many physiological states and metabolic diseases, such as obesity and IR [5–8]. However, the connections between obesity, myokine secretion, and metabolic dysfunction remain to be elucidated. Among myokines, musclin is a newly discovered, 130-amino acid peptide that was first reported by Nishizawa et al. Musclin has been found to be almost exclusively expressed in skeletal muscles [9]. Musclin is a pleiotropic myokine that is involved in the regulation of energy homeostasis. Nishizawa et al. reported that musclin mRNA expression was augmented in the skeletal muscle of obese mice [9]. In vitro studies have demonstrated that musclin significantly inhibited insulin-stimulated 2-deoxy-D-[1-3H]-glucose (2-DG) uptake and glycogen synthesis [9]. Furthermore, Liu et al. reported that pre incubating muscles with musclin reduced protein kinase B activation in the insulin-signaling cascade [10]. Although musclin has been shown to be a novel, endogenous obesity-related factor in animal models, the mechanism of its bioactivity and its expression in humans remain largely unknown.

In this study, we investigated the circulating levels of musclin and the clinical parameters associated with musclin levels in subjects from a human cohort with or without obesity. Furthermore, a high-fat diet (HFD) rat obesity model was used to test the hypothesis that musclin plays a role in obesity-associated IR in skeletal muscle.

## Methods

### Animals and reagents

Male Sprague–Dawley (SD) rats (155 ± 5 g) were provided by the Animal Department, Peking University Health Science Center. All animal care and experimental protocols complied with the Animal Management Rules of the People’s Republic of China (Ministry of Health, P.R. China, document no. 55, 2001) and the Care and Use of Laboratory Animals published by the US National Institutes of Health (NIH Publication No. 85-23, revised 1996) and were approved by the Animal Care Committee of Health Science Center, Peking University. Musclin peptide was synthesized by Phoenix Pharmaceuticals (Belmont, CA, USA). [^3^H] Deoxy-glucose was obtained from PerkinElmer (Boston, MA, USA), and insulin was obtained from Sigma-Aldrich (St. Louis, MO, USA). Antibodies against GRP78, GRP94 were from Abcam (Cambridge, UK). Antibodies against musclin and β-actin and all secondary antibodies were obtained from Santa Cruz Biotechnology (Santa Cruz, CA, USA). Antibody against GLUT-4 was from Wuhan BOSTER Bioengineering (Wuhan, Hubei, China).

### Preparation of the animal model

Male SD rats (155 ± 5 g) were randomly divided into the 2 following groups (*n* = 8 each): the normal diet (ND) group, comprising 14,610 kJ/kg and energy contents (%) for carbohydrates, fat, and protein of 66.50, 10.21, and 23.29, respectively; and the HFD group, comprising 19,315 kJ/kg, 200 g fat/kg (170 g of lard +30 g of corn oil to provide essential fatty acids) and 1% cholesterol by weight plus normal drinking water. The HFD was formulated to provide 40% of the total energy from fat by replacing carbohydrate energy with lard and corn oil energy. The HFD had the same amounts of vitamins and minerals per kilojoule as the ND [11]. After 2 days of further treatment, the rats were anesthetized with urethane (1 g/kg, intraperitoneally) at the end of a dark cycle and overnight fasting and were then euthanized. Blood was collected in a heparinized syringe from the abdominal aorta and mixed with 1 mg/mL EDTA-2Na and 500 KIU/mL aprotinin or heparin. Plasma and serum were obtained by centrifugation at 3000 rpm for 10 min at 4 °C and stored at −70 °C. White gastrocnemius muscles were collected and weighed. All tissue samples were frozen in liquid nitrogen.

### Subjects

The study was performed in accordance with the Helsinki Declaration and was approved by the Ethics Committee of the Second Affiliated Hospital of Harbin Medical University, Harbin, China. Written informed consent was obtained from all participants prior to participation. We selected 117 consecutive subjects from the general population who had undergone medical check-ups at the Outpatient Department of the Second Affiliated Hospital of Harbin Medical University. Exclusion factors were diseases affecting the metabolic state or being unsuitable for participation in this study. All subjects completed a self-administered questionnaire to provide data on age, smoking history, alcohol consumption, history of T2DM and medications. All subjects underwent a physical examination, including measurements of height and weight (while wearing light clothes and no shoes), body mass index (BMI, kg/m^2^), and blood pressure. Waist circumferences were calculated midway between the iliac crest and rib cage and were rounded to the nearest 0.1 cm;hip circumference was measured at the point of the maximum extension of the buttocks. Waist-to-hip ratios were calculated by dividing the waist circumference (cm) by the hip circumference (cm). According to the recommendations of the Working Group on Obesity in China, subjects with BMI between 18.5 and 24.0 kg/m^2^ were considered normal, while those with BMIs between 24.0 and 28.0 kg/m^2^ or over 28.0 were defined as overweight or obese [12].

### Radioimmunoassays of musclin levels in muscle and plasma

Musclin levels were measured using a specific, commercially available radioimmunoassay kit (Beijing Sino-UK Institute of Biological Technology, Beijing, China). Gastrocnemius muscles were immediately acidified by the addition of 1.0 mol/L acetic acid and were then heated at 100 °C for 10 min to inactivate proteases. Tissue homogenates were prepared using a Polytron homogenizer, centrifuged at 17,000×g for 20 min, and then the supernatants collected. The plasma samples were pre-treated with aprotinin (500 KIU/mL). The assay sensitivity provided by the kit manufacturer was 1.25 pg/mL, and the standard curve range was from 0 to 400 pg/mL. The assays showed a good degree of parallelism. The intra- and interassay coefficients of variation were validated in the present study and were 7.1 and 10%, respectively. There was no cross-reactivity with rat IL-15, insulin-like growth factor-1, platelet-derived growth factor, fibroblast growth factor, transforming growth factor-β or hepatocyte growth factor.

### Glucose uptake

After fasting overnight, rats were anesthetized using urethane (1 g/kg, intraperitoneally) and euthanized; the soleus muscles were obtained by dissection (25-30 mg) and preincubated in 12-well plates at their resting length. The soleus muscles were then incubated in 2 mL of Krebs-Henseleit buffer (KHB) containing 40 mmol/L mannitol, 0.1% bovine serum albumin (BSA) and 8 mmol/L glucose with or without 2 mIU/mL insulin at 37 °C for 2 h. The muscles were then transferred to 12-well plates in 2 mL of KHB containing 40 mmol/L mannitol, 0.1% BSA, 1.5 μCi/ml [^3^H]-2-deoxy-D-glucose and insulin at the same concentration as was used during the preceding incubation. The plates were placed in a water bath at 37 °C under continuous shaking (60 beats/min) and bubbling of 95% O_2_ and 5% CO_2_. After 30 min, the muscles were placed in scintillation vials containing 100 μL of formic acid and 100 μL of 30% hydrogen peroxide and were counted in a Packard liquid scintillation counter with channels preset for simultaneous ^3^H [13, 14].

### Muscle preparation and incubation

After an overnight food restriction, the rats were anesthetized by urethane (1 g/kg, intraperitoneally) and euthanized. The soleus muscles were dissected (25-30 mg) with a bistoury and were preincubated for 30 min in 12-well plates at their resting length in KHB with 2% BSA. After preincubation, muscles were incubated in KHB with or without 1.5 × 10^−7^ mol/L musclin for 3 h, as previously described. The plates were placed in a water bath at 37.8 °C under continuous shaking (60 beats/min) and bubbling with 95% O_2_ and 5% CO_2_. After co-incubation for 3 h, the tissues were rapidly blotted on ice-cold filter paper and stored at −80 °C until the Western blot analysis.

### Real-time PCR analysis

Total RNA from the gastrocnemius muscles was isolated and reverse transcribed using a reverse transcription system (Promega, Madison, WI, USA). In total, 20 μL of the reaction mixture underwent real-time PCR. The amount of PCR product formed in each cycle was evaluated by SYBR Green I fluorescence. The rat primers used for musclin wereforward, 5′-GGT GTC CTTG GAGA ATGATG-3′ and reverse, 5′-CGGTTTCTACCAATTCGATC -3′, and those for β-actin were forward, 5′ – A TCTGGCACC ACA CCTTC-3′ and reverse, 5′-AG CCAGGTCCAGAC G CA-3′. All amplification reactions used the Mx3000 Multiplex Quantitative PCR System (Stratagene, La Jolla, CA, USA). After denaturation at 95 °C for 7 min, the solution was subjected to PCR for musclin at 95 °C for 30 s, 58 °C for 30 s, and 72 °C for 40 s for 45 cycles.

### Western blot analysis

Skeletal muscle tissues were homogenized in lysis buffer [0.1 mol/L NaCl, 0.01 mol/L Tris–HCl (pH 7.5), 1 mmol/L EDTA, 1 mmol/L PMSF, 1%TritonX-100, 10 μg/ml pepstatin A and 500 KIU/mL aprotinin], and the homogenates were centrifuged at 3000 rpm for 15 min at 4 °C. Protein samples were separated using 10% SDS-PAGE and were then transferred to nitrocellulose for 3 h at 4 °C. The membranes were blocked with 5% nonfat, dried milk for 1 h at room temperature and were then incubated with the primary antibodies anti-β-actin, anti-GRP78, anti-GRP94, anti-musclin or anti-glucose transporter type-4 (GLUT-4) overnight at 4-8 °C. Then,the membranes were incubated for 1 h with the secondary antibodies (horseradish peroxidase-conjugated anti-mouse or anti-rabbit IgG). Protein expression was analyzed use NIH image analysis software (Bethesda, Maryland, USA) and normalized to β-actin expression. All experiments were repeated at least 3 times.

### Statistical analysis

The data were analyzed using SPSS 20.0 (SPSS Inc., Chicago, IL, USA), and the results are expressed as the mean ± standard deviation. For continuous variables, comparisons between 2 groups were made using an unpaired Student t test, and those among more than 2 groups were made using one-way ANOVA, followed by Newman–Keuls multiple comparison test. Skewed data were analyzed using the Mann–Whitney U and Kruskal–Wallis H tests. Correlations between variables were determined using a simple linear regression analysis (Spearman’s correlation). Unadjusted and adjusted odds ratios (ORs) with 95% confidence intervals (CIs) predictive of subjects with overweight/obesity based on musclin level were generated using univariate and multivariate logistic regression analyses after controlling for other potential covariates. Values of *P <* 0.05 were considered significant.

## Results

### Subjects’characteristics

Demographic and laboratory characteristics stratified by BMI are presented in Table 1. Compared with the lean subjects, the individuals with overweight/obesity had higher diastolic blood pressure(DBP),TG,fasting serum insulin and HOMA-IR levels. There were no significant differences between the two groups in the other parameters, including age, fasting plasma glucose (FPG), TC, LDL-C and HDL-C.

**Table 1:** Subjects’ characteristics

Variable	Lean (*n* = 44)	Overweight/obese (*n* = 73)	*P* value
Age (years)	51.75 ± 13.8	47.89 ± 9.8	0.08
Male/female	21(47.73%)/23	48(65.75%)/25	0.055
T2DM history	28(63.64%)	48(65.75%)	0.816
Current smoker (%)	9(20.45%)	26(35.62%)	0.083
Alcohol use (%)	14(31.82%)	31(42.47%)	0.252
Waist-to-hip ratio	0.9 ± 0.04	0.9 ± 0.05	0.491
Systolic blood pressure (mmHg)	127.36 ± 17.73	132.05 ± 16.73	0.154
Diastolic blood pressure (mmHg)	82.59 ± 9.43	87.68 ± 9.02	0.004^**^
Total cholesterol (mmol/L)	5.06 ± 1.01	5.36 ± 1.16	0.155
Triglycerides (mmol/L)	1.69 ± 1.41	2.55 ± 2.24	0.025*
High-density lipoprotein (mmol/L)	1.33 ± 0.29	1.26 ± 0.27	0.157
Low-density lipoprotein (mmol/L)	3.07 ± 0.84	3.2 ± 0.81	0.407
Fasting plasma glucose (mmol/L)	7.28 ± 3.29	8.18 ± 3.7	0.186
Fasting serum insulin (mmol/L)	8.46 ± 5.92	13.44 ± 8.70	0.001^**^
HOMA-IR (μIU x mol/L)	3.02 ± 3.94	5.42 ± 5.19	0.009^**^
Hemoglobin A_1c_ (%)	6.94 ± 2.32	7.54 ± 2.28	0.173
Musclin(ng/L)	80.39 ± 16.35	89.49 ± 19.00	0.009^**^

Data are presented as the mean ± standard deviation or as proportions (%)

*P < 0.05 vs. controls, **P < 0.01 vs. controls

*T2DM* type 2 diabetes mellitus, *HOMA-IR* homeostasis model assessment of insulin resistance

### Elevated plasma musclin levels in subjects with overweight/obesity

**Fig. 1:**
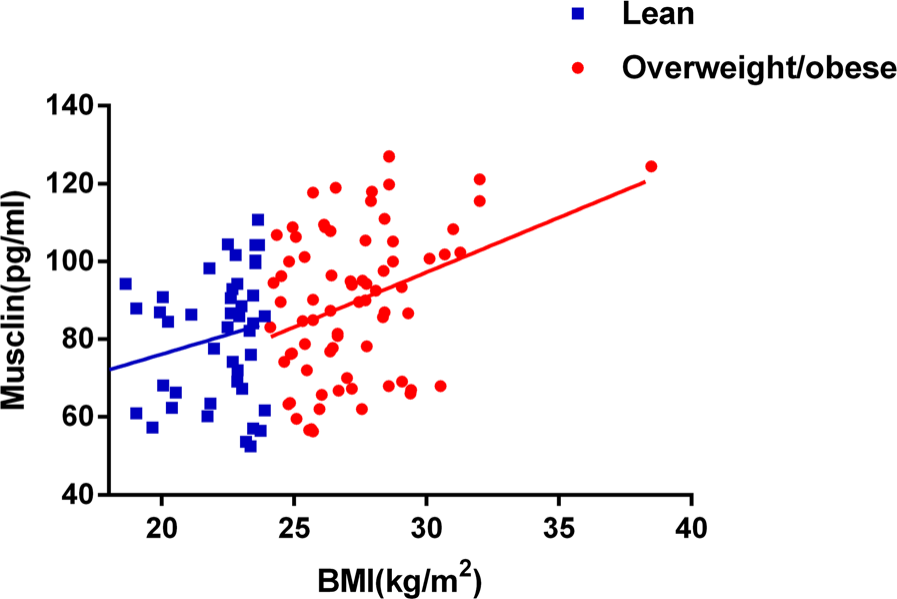
Comparison of plasma musclin levels in lean controls and subjects with overweight/obesity

The musclin data were normally distributed. We found higher plasma musclin levels in subjects with overweight/obesity than in lean subjects (89.49 ± 19.00 ng/L vs 80.39 ± 16.35 ng/L, *P* < 0.01) (Fig. 1 and Table 1).

### Relation between plasma musclin levels and overweight/obesity parameters

**Fig. 2:**
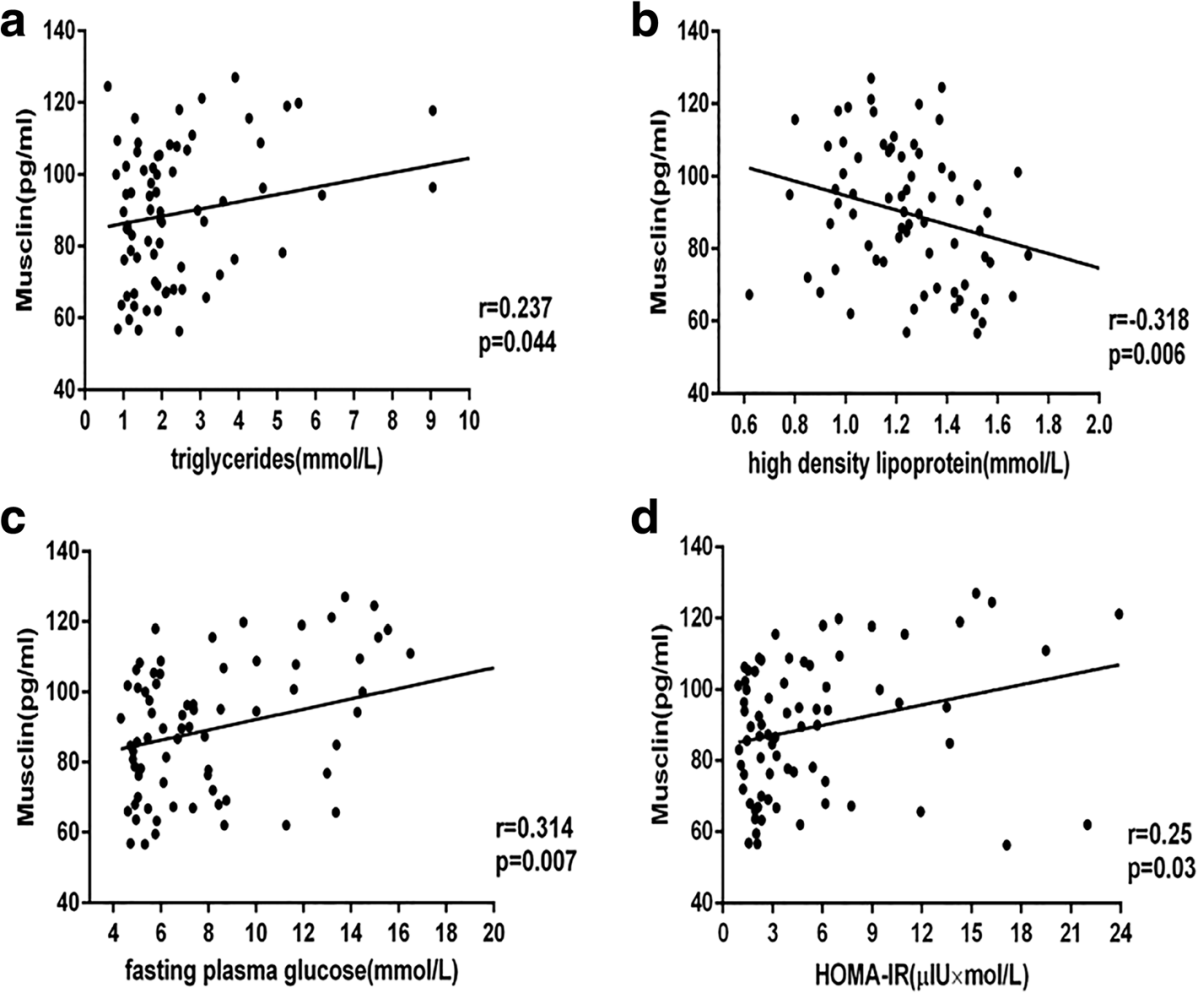
Relation between plasma musclin levels and overweight/obesity parameters. HOMA-IR: homeostasis model assessment of insulin resistance. r = correlation coefficient *P < 0.05, **P < 0.01

The Spearman correlation coefficients for associations between musclin concentrations and other parameters are summarized in Table 2. In all subjects, plasma musclin levels were significantly positively associated with BMI(*r* = 0.327, *P* < 0.01) and TG(*r* = 0.191, *P* < 0.05) but negatively associated with HDL(*r* = −0.282, *P* < 0.01). In lean subjects, plasma musclin levels were not associated with BMI, DBP, SBP, or the other variables mentioned above. However, in subjects with overweight/obesity, plasma musclin levels were significantly positively associated with BMI (*r* = 0.287, *P* < 0.05) (Fig. 1), TG (*r* = 0.237, *P* < 0.05), FPG (*r* = 0.314, *P* < 0.01) and HOMA-IR (*r* = 0.250, *P* < 0.05) levels but negatively associated with HDL (*r* = −0.318, *P* < 0.05) (Fig. 2).

**Table 2:** Correlations of plasma musclin with anthropometric parameters

Variable	Total (*n* = 117)		Lean (*n* = 44)		Overweight/obese (*n* = 73)	
	*r*	*P*	*r*	*p*	*r*	*p*
Body mass index (kg/m^2^)	0.327	0.001**	0.142	0.356	0.287	0.014*
Waist-to-hip ratio	−0.077	0.410	−0.050	0.746	−0.150	0.204
Systolic blood pressure (mmHg)	−0.097	0.299	−0.080	0.607	−0.167	0.157
Diastolic blood pressure (mmHg)	0.009	0.926	−0.006	0.967	−0.080	0.499
Total cholesterol (mmol/L)	−0.009	0.924	−0.202	0.188	0.044	0.709
Triglycerides (mmol/L)	0.191	0.039*	−0.019	0.903	0.237	0.044*
High-density lipoprotein (mmol/L)	−0.282	0.002**	−0.150	0.331	−0.318	0.006**
Low-density lipoprotein (mmol/L)	−0.120	0.197	−0.132	0.394	−0.156	0.189
Fasting plasma glucose (mmol/L)	−0.158	0.088	−0.174	0.258	0.314	0.007**
Fasting serum insulin (mmol/L)	0.089	0.342	−0.199	0.194	0.147	0.215
HOMA-IR (μIU x mol/L)	0.13	0.164	−0.292	0.054	0.25	0.033*
Hemoglobin A_1c_ (%)	0.028	0.766	−0.300	0.052	0.172	0.146

Correlations were determined using Spearman correlations

*HOMA-IR* homeostasis model assessment of insulin resistance. *P < 0.05, **P < 0.01

### Correlation between overweight/obesity and the musclin level

The ORs of the musclin level being predictive of subjects with overweight/obesity were determined using univariate and multivariate logistic regression analyses; the results are shown in Table 3. In the univariate logistic regression analyses, the OR and 95% CI were 1.029(1.006-1.051) (*P* = 0.011). In model 1 of the multivariate logistic regression analyses, after adjusting for age and gender, the OR and 95% CI were 1.027(1.005-1.051) (*P* = 0.017). In model 2, after adjusting for age, gender, SBP, LDL-C, TC, insulin and HbA1c, the OR and 95% CI were 1.032(1.006-1.059) (*P* = 0.015). In model 3, after adjusting for age, gender, SBP, LDL-C, HDL-C, TC, TG, insulin, HbA1c and FPG, the OR and 95% CI were 1.033(1.005-1.061) (*P* = 0.019). In model 4, after adjusting for age, gender, SBP, LDL-C, HDL-C, TC, TG, insulin, HbA1c, FPG, smoking and drinking history, the OR and 95% CI were 1.031(1.003-1.060) (*P* = 0.031);

**Table 3:** Univariate and multivariate logistic regression analyses of musclin predicting overweight/obese patients (*n* = 117)

Variable	*P*	OR	95% CI
Univariate	0.011	1.029	1.006-1.051
Multivariate			
Model 1^a^	0.017	1.027	1.005-1.051
Model 2^b^	0.015	1.032	1.006-1.059
Model3^c^	0.019	1.033	1.005-1.061
Model4^d^	0.031	1.031	1.003-1.060

Odds ratios(ORs) for overweight/obesity were calculated by logistic regression models. *CI* confidence interval

^a^Adjusted for age and gender

^b^ Adjusted for age, gender, systolic blood pressure, low-density lipoprotein, total cholesterol, insulin and hemoglobin A_1c_

^c^Adjusted for age, gender, systolic blood pressure, low-density lipoprotein, high-density lipoprotein,total cholesterol, triglycerides, insulin, hemoglobin A_1c_, and fasting plasma glucose

^d^ Adjusted for age, gender, systolic blood pressure, low-density lipoprotein, high-density lipoprotein,total cholesterol, triglycerides, insulin, hemoglobin A_1c_, fasting plasma glucose, smoking and drinking history

### Insulin sensitivity was impaired in HFD rats

**Fig. 3:**
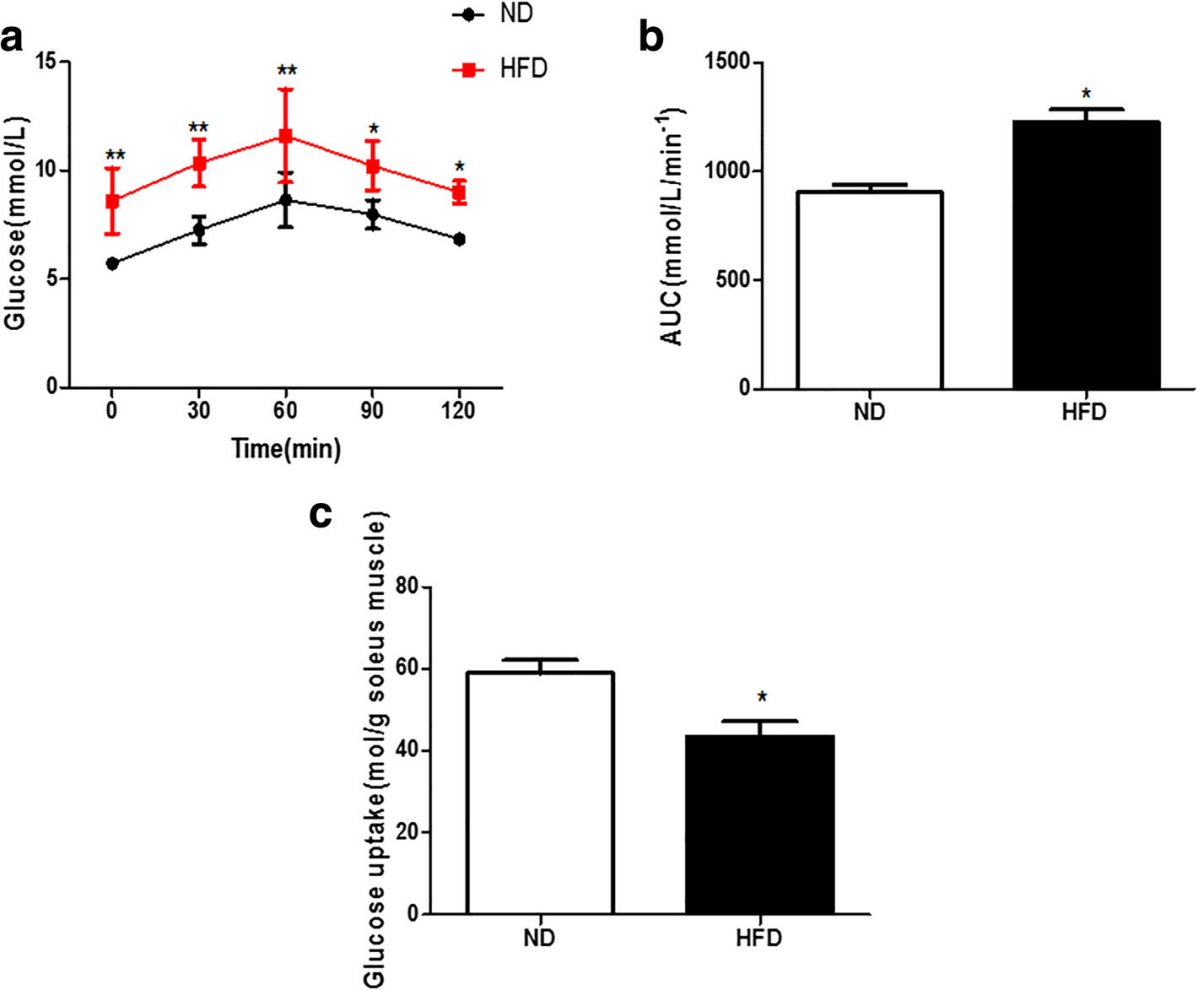
Twenty-week high-fat diet induced insulin resistance. **a**: Mean blood glucose levels during the oral glucose tolerance test; **b**: Total area under the glucose curve. **c**: Effect of high-fat diet on glucose uptake in soleus muscles. ND: normal diet; HFD: high-fat diet; AUC: area under the curve. Values are the mean ± standard error of the mean; (*n* = 8); *P < 0.05 vsND,**P < 0.01 vs ND

After 20 weeks, the body weight of the HFD rats increased by 13.49% (*P* < 0.01), the Lee index increased by 4.88% (*P* < 0.01) and the fat mass/body weight (%) increased by 89.32% (*P* < 0.01) (Table 4). The 20-weeks HFD also increased TC (by 24.79%), TG (by 27.78%) and LDL (by 49.60%) levels(all *P* < 0.05) but decreased the HDL level by 52.78% (*P* < 0.01) (Table 4). Insulin sensitivity was impaired in HFD rats, as shown by the HOMA-IR level,which increased by 113.45% (*P* < 0.01) (Additional file 1: Table S1). Compared with the control rats, the HFD increased serum insulin and FBG levels in rats by 76.68% (*P* < 0.01) and 37.6%(*P* < 0.01), respectively (Table 4). The glucose response during the OGTT in the HFD group was markedly increased by 50% (*P* < 0.01), 43% (*P* < 0.01), 34% (*P* < 0.01), 28% (*P* < 0.05) and 31% (*P* < 0.05) at the 0, 30, 60, 90, and 120 min time points, respectively, compared with those in the ND group (Fig. 3a). The total area under the glucose curve was also significantly increased by 36% (*P* < 0.05) in the HFD group (Fig. 3b). To accurately examine IR, we measured insulin-induced 2-DG uptake in the soleus muscles. In the HFD rats, 2-DG uptake (glucose transport activity) showed a 26.3% (*P* < 0.05) decrease compared with that in the ND rats (Fig. 3c).

**Table 4:** Comparison of plasma biochemical indicators in HFD and ND rats

Parameters	ND	HFD
Body weight (g)	546.30 ± 16.29	620.01 ± 13.31^**^
Lee index	301.27 ± 1.73	315.97 ± 2.26^**^
Fat mass/body weight (%)	4.12 ± 0.29	7.80 ± 0.72^**^
Triglyceride (mmol/L)	0.72 ± 0.04	0.92 ± 0.05^*^
Total cholesterol (mmol/L)	2.42 ± 0.10	3.02 ± 0.19^*^
High-density lipoprotein (mmol/L)	1.08 ± 0.10	0.51 ± 0.03^**^
Low-density lipoprotein (mmol/L)	1.25 ± 0.11	1.87 ± 0.22^*^
Fasting serum insulin(uIU/mL)	11.32 ± 0.46	20.0 ± 1.77^**^
Fasting blood glucose(mmol/L)	5.46 ± 0.09	7.51 ± 0.28^**^
HOMA-IR (μIU x mol/L)	2.75 ± 0.14	5.87 ± 0.67^**^

*HFD* high-fat diet, *HOMA-IRI* homeostasis model assessment of insulin resistance, *ND* normal diet. The data are shown as the mean ± standard error of the mean of each group; (*n* = 8); ^*^ *P* < 0.05 vs ND group. ^**^ *P* < 0.01 vs ND group

### Musclin expression and its circulating levels increased in HFD rats

**Fig. 4:**
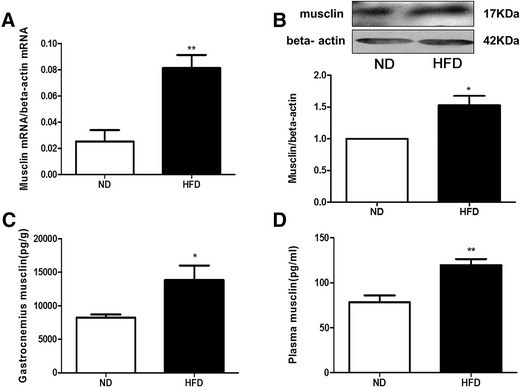
Musclin expression and its circulating musclin levels increased in HFD rats. **a**: Real-time PCR analysis of musclin expression in gastrocnemius muscles. **b**: Western blot analysis of musclin protein expression in gastrocnemius muscles and ratio of musclin to β-actin determined by quantitative analysis. **c**: Radioimmunoassay analysis of musclin content in gastrocnemius muscles. **d**: Radioimmunoassay analysis of plasma musclin levels. ND: normal diet; HFD: high-fat diet. Values are the mean ± standard error of the mean; (*n* = 8); *P < 0.05 vs ND

In the rat gastrocnemius muscles, the HFD increased musclin mRNA expression by 220% (*P* < 0.01) (Fig. 4a) and increased the musclin protein level by 52.8% (*P* < 0.05) and 67.70% (8.25 ± 0.45 ng/g vs 13.84 ± 2.16 ng/g,*P* < 0.05), as determined by Western blot and radioimmunoassay analyses, respectively (Fig. 4b,c). Compared with the control group, musclin immunoreactivity (musclin-ir) in plasma was increased in HFD rats (78.34 ± 7.52 ng/L vs 119.6 ± 6.71 ng/L,*P* < 0.01) (Fig. 4d).

### Correlation between musclin content and an indicator of insulin resistance in HFD rats

**Fig. 5:**
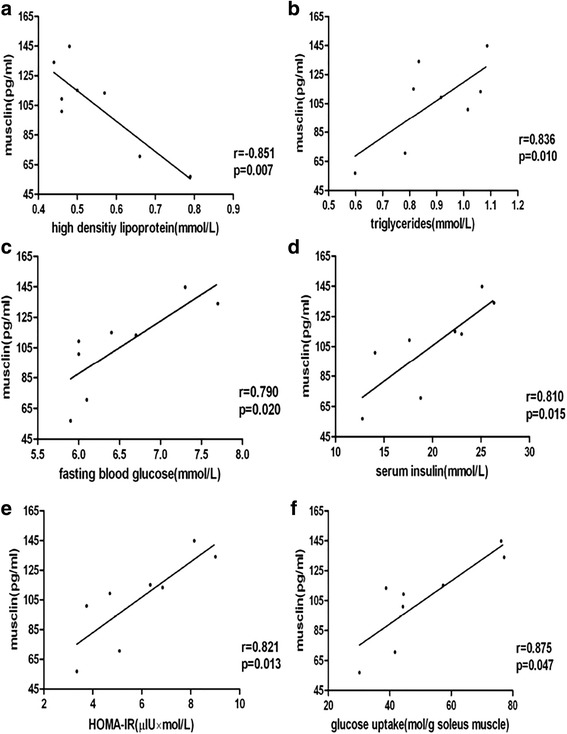
Correlation between plasma immunoreactive musclin concentrations and plasma biochemical indicator in HFD rats. HOMA-IR: homeostasis model assessment of insulin resistance. r = correlation coefficient *P < 0.05, **P < 0.01

**Fig. 6:**
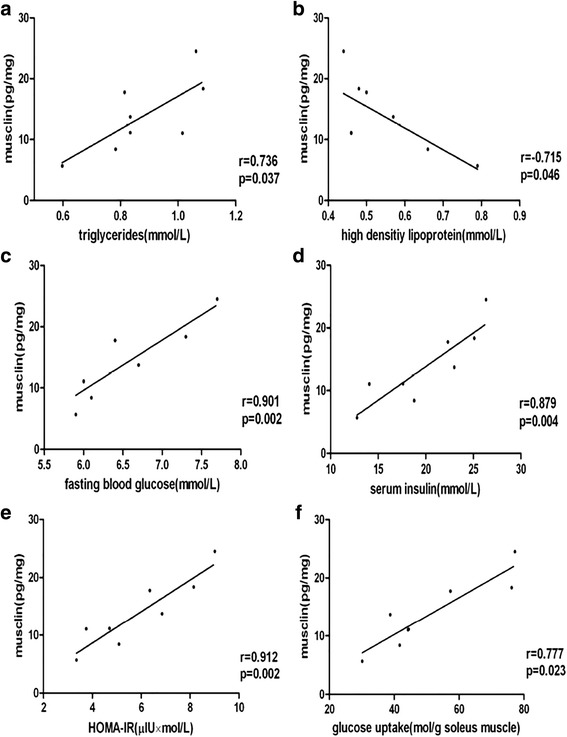
Correlation between skeletal muscle immunoreactive musclin concentrations and plasma biochemical indicator in HFD rats. HOMA-IR: homeostasis model assessment of insulin resistance. r = correlation coefficient *P < 0.05, **P < 0.01

The plasma musclin-ir concentrations were positively correlated with FBG (*r* = 0.79, *p* < 0.05), serum insulin (*r* = 0.81, *p* < 0.05) and glucose uptake (*r* = 0.875, *p* < 0.05) of skeletal muscle in HFD rats (Fig. 5 and Additional file 1: Table S1). There was no significant correlation between plasma musclin content and obese indicators including weight, Lee index and fat mass/body weight. The skeletal muscle musclin-ir displayed a positive correlation with FBG (*r* = 0.901, *p* < 0.01), serum insulin (*r* = 0.879, *p* < 0.01) and glucose uptake (*r* = 0.777, *p* < 0.05) of skeletal muscle in HFD rats (Fig. 6 and Additional file 2: Table S2). There was no significant correlation between skeletal muscle musclin content and obese indicators including weight, Lee index and fat mass/body weight.

### Musclin induced IR and inhibited the protein expression of GLUT-4 in rat skeletal muscle

**Fig. 7:**
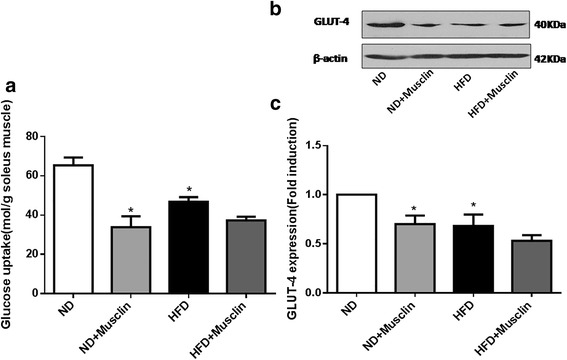
Incubating soleus muscles with musclin induced IR and inhibited the protein expression of GLUT-4. **a**: Glucose transport activity (2-DG) in soleus muscles. **b**: Western blot analysis of the protein level of 40-kDa GLUT-4 in soleus muscles. **c**: Quantitative analysis of GLUT-4 protein expression. GLUT-4:glucose transporter type 4. Values are the mean ± standard error of the mean; (*n* = 3); *P < 0.05 vsND,**P < 0.01 vs ND

To determine whether musclin impaired the glucose uptake ability of skeletal muscle, we tested the effect of musclin on 2-DG uptake in the soleus muscles. We observed a 48.3% (*P* < 0.01) decrease in 2-DG uptake in the ND group after incubation with musclin (1.5 × 10^−7^ mol/L) compared with the control, while there was no significant decreasein 2-DG uptake in the HFD group after incubation with musclin (Fig. 7a). Compared with the ND rats, the HFD rats exhibited a marked decrease in the GLUT-4 protein level (*P* < 0.05). The protein expression of GLUT-4 in the soleus muscle was significantly downregulated by 30% (*P* < 0.05) in the ND group after incubation with musclin compared with the control (Fig. 7b,c).

### Musclin increased skeletal muscle endoplasmic reticulum stress (ERS) marker levels

**Fig. 8:**
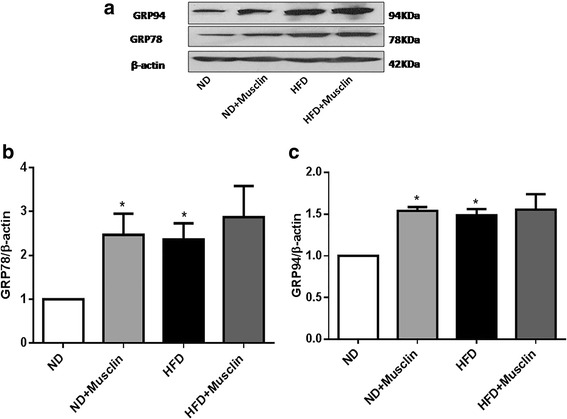
Effect of musclin on ERS markers in rat soleus muscles. **a**: Western blot analysis of the protein levels of the 78-kDa glucose-regulated protein (GRP78) and the 94-kDa GRP94 in soleus muscles. **b**: Quantitative analysis of GRP78 protein expression. **c**: Quantitative analysis of GRP94 protein expression. Values are the mean ± standard error of the mean; (*n* = 3); *P < 0.05 vsND,**P < 0.01 vs ND

After 20 weeks, the HFD rats showed GRP78 and GRP94 protein levels in skeletal muscle that were significantly increased by 136.1 and 48.6% (both *P* < 0.05), respectively, compared with the ND rats. The Western blot analysis showed thatinthe ND group, muscle incubation with musclin increased the protein levels of GRP78 and GRP94 by 146.8 and 54% (both *P* < 0.05), respectively. However, in the HFD group, there were no differences in GRP78 and GRP94 protein expression after the musclin incubation (Fig. 8).

## Discussion

The present study demonstrated for the first time that plasma musclin levels were significantly higher in subjects with obesity than in lean controls. In addition, the plasma musclin concentration was positively correlated with IR-related laboratory parameters as well as fasting glucose and HOMA-IR levels. Furthermore, the human study results were supported by the findings of the animal experiments, which showed that rats with obesity-associated IR had markedly increased plasma and skeletal muscle musclin expression. Using ex-vivo assays, we also found that musclin impaired insulin-induced glucose uptake and inhibited the protein expression of GLUT-4 related to the activation of ERS. This alteration of endogenous musclin expression in skeletal muscle and plasma in humans and rats with obesity-associated IR has never been previously reported.

Obesity-induced IR is a key pathophysiological feature of T2DM; however, the specific mechanism linking IR and obesity has not been established before. In our study, plasma musclin levels were investigated in subjects with overweight/obesity (BMI > 24 kg/m^2^), and we demonstrated for the first time that musclin plasma concentrations were significantly increased in the subjects with overweight/obesity compared with the lean subjects. Our data showed that the subjects with overweight/obesity had higher insulin and HOMA-IR levels, suggesting that the subjects with overweight/obesity had obtained IR. In addition, we found that the musclin levels in the subjects with overweight/obesity were significantly correlated with markers of obesity and IR, such as BMI, HOMA-IR and FBG levels. Hence, we speculate that musclin may play an important role in obesity-associated IR.

We then performed a further study in rats with obesity-associated IR induced by a HFD. After 20 weeks, the HFD rats exhibited dyslipidemia, as indicated by higher serum TC and TG levels, similar to what is observed in human obesity. IR leads to hyperinsulinemia and a decreased glucose metabolism [15]. Our results also show that the obese rats exhibited characteristics of IR, such as elevated blood glucose levels, hyperinsulinemia and impaired glucose tolerance. Additionally, the glucose uptake by skeletal muscle with IR was significantly decreased, which is in agreement with the findings of previous reports [16]. Recent research has shown that myofibers produce and release musclin in a fiber-type-specific manner, wherein higher levels of musclin are found in the fast-twitch plantaris and white gastrocnemius muscles, especially in the type IIb fibers [17]. In the present study, we used white gastrocnemius rat musclesto to investigate the expression of musclin. We found that the gene expression of musclin was clearly upregulated in the HFD rat skeletal muscles, and the musclin protein levels were also significantly increased, as determined by Western blot and radioimmunoassay analyses. Several studies have demonstrated increased musclin levels is associated with metabolic disorder. Nishizawa et al. reported that musclin mRNA expression was augmented by approximately 4-fold in the gastrocnemius muscles of obese KKAy mice and db/db mice [9]. Yu et al. found musclin expression was significantly elevated in the skeletal muscle of HFD rats [18]. Chen et al. reported increased circulating levels of musclin in newly diagnosed T2DM patients [19]. In our study, we demonstrated for the first time that the plasma musclin-ir was up regulated in rats with obesity-associated IR. We then determined the causes of the higher musclin plasma levels in HFD rats. Compared with the ND rats, the HFD rats showed skeletal muscle musclin mRNA expression that was increased by 2-fold. In addition, in the HFD group, the musclin expression in the skeletal muscles was approximately 100-fold higher than that in the plasma. Based on a previous report demonstrating that musclin is exclusively expressed in skeletal muscles [9], together with the significantly higher musclin expression in skeletal muscles, the findings of the current study suggest that the increased plasma musclin in rats with IR might be secreted predominantly by skeletal muscles.

A few investigations have addressed the mechanism and regulation of musclin expression in the state of IR. Our results suggested that incubating the skeletal muscles of ND rats with musclin could induce IR, leading to decreased 2-DG uptake. A recent study has shown that the preincubation of skeletal muscles with musclin caused decreased insulin-stimulated 2-DG uptake and decreased Akt/PKB activation in the insulin-signaling cascade [10]. In our study, we also found that musclin suppresses the expression of GLUT-4 protein in ND rats. These results suggest that musclin could exert effects on glucose homeostasis that may be mediated via changes in the insulin sensitivity of skeletal muscle.

Notably, our study demonstrated that musclin caused ERS in skeletal muscle. In recent years, ERS has been implicated in the development of peripheral IR, obesity and T2DM [20]. Deldicque et al. showed that a 20-weeks HFD increased the protein and mRNA levels of factors involved in the unfolded protein response, such as binding protein/GRP78, p-protein kinase R-like ER protein kinase, CHOP and inositol-requiring enzyme 1ɑ in skeletal muscle [21]. Gu et al. found that palmitate can induce a high expression of musclin in C2C12 myotubes, and that the PERK signaling pathway is potentially involved in this process [22]. In our study, we also found that ERS markers, such as GRP78 and GRP94,were markedly increased in the skeletal muscles of the HFD rats, which was consistent with the findings of previous studies. Furthermore, we also found that musclin incubation increased the protein levels of GRP78 and GRP94.Therefore, these results demonstrated that musclin could induce IR, at least in part, through causing ERS in skeletal muscles.

## Conclusion

In summary, we showed that HFD upregulated the expression of endogenous musclin in skeletal muscles and plasma in obesity-associated IR. Treating skeletal muscle with musclin induced IR and impaired the glucose metabolism, at least in part, through causing ERS. Musclin is an important myokine that participates in the development of skeletal muscle IR and the regulation of peripheral glucose homeostasis in subjects with obesity and HFD rats. Further research is warranted to investigate the functional role of musclin in the development of obesity-associated IR and its mechanism of regulation.

## Additional files

Additional file 1: Table S1. Correlation between plasma immunoreactive musclin concentrations and plasma biochemical indicator in rats. (DOC 27 kb)

Additional file 2: Table S2. Correlation between skeletal muscle immunoreactive musclin concentrations and plasma biochemical indicator in rats. (DOC 29 kb)

## Abbreviations

2-DG: 2-deoxy-D-[1-3H]-glucose
DBP: Diastolic blood pressure
FPG: Fasting plasma glucose
GRP: Glucose-regulated protein
HFD: High-fat diet
IL-6: Interleukin-6
IR: Insulin resistance
LDL: Low-density lipoprotein cholesterol
ND: Normal diet
OGTT: Oral glucose tolerance test
SD: Sprague–Dawley
T2DM: Type 2 diabetes mellitus
TC: total cholesterol
TG: Triglyceride
