# Supplementary material for: Positive association between musclin and insulin resistance in obesity: evidence of a human study and an animal experiment
Source: Nutr Metab (Lond). 2017 Jul 10;14:46. doi: 10.1186/s12986-017-0199-x (PMC5504815; doi:10.1186/s12986-017-0199-x)
Supplement: Supplementary file 1 — Correlation between plasma immunoreactive musclin concentrations and plasma biochemical indicator in rats. (DOC 27 kb) [file 12986_2017_199_MOESM1_ESM.doc]

**Table S1** Correlation between plasma immunoreactive musclin concentrations and plasma biochemical indicator in rats

| Parameters | ND | | | HFD | |
| --- | --- | --- | --- | --- | --- |
|  | r | P | r | | P |
| Body weight | 0.320 | 0.439 | 0.079 | | 0.867 |
| Lee index | 0.401 | 0.430 | -0.003 | | 0.995 |
| Fat mass/Body weight (%) | -0.365 | 0.477 | 0.344 | | 0.505 |
| Triglyceride (mmol/L) | 0.013 | 0.977 | 0.836 | | 0.010^*^ |
| Total cholesterol (mmol/L) | 0.375 | 0.359 | 0.599 | | 0.116 |
| High density lipoprotein (mmol/L) | 0.363 | 0.377 | -0.851 | | 0.007^**^ |
| Low density lipoprotein (mmol/L) | 0.247 | 0.555 | 0.564 | | 0.146 |
| Fasting serum insulin(uIU/mL) | 0.230 | 0.584 | 0.810 | | 0.015^*^ |
| Fasting blood glucose(mmol/L) | 0.428 | 0.290 | 0.790 | | 0.020^*^ |
| Glucose uptake(mol/g soleus muscle) | 0.011 | 0.980 | 0.875 | | 0.047^*^ |
| HOMA-IRI (µIU x mol/L) | 0.326 | 0.431 | 0.821 | | 0.013^*^ |

HOMA-IRI: homeostasis model assessment insulin resistance index. r = correlation coefficient (n = 8) ^*^P< 0.05, ^**^P<0.01
