# Supplementary material for: Positive association between musclin and insulin resistance in obesity: evidence of a human study and an animal experiment
Source: Nutr Metab (Lond). 2017 Jul 10;14:46. doi: 10.1186/s12986-017-0199-x (PMC5504815; doi:10.1186/s12986-017-0199-x)
Supplement: Supplementary file 2 — Correlation between skeletal muscle immunoreactive musclin concentrations and plasma biochemical indicator in rats. (DOC 29 kb) [file 12986_2017_199_MOESM2_ESM.doc]

**Table S2** Correlation between skeletal muscle immunoreactive musclin concentrations and plasma biochemical indicator in rats

| Parameters | ND | | HFD | |
| --- | --- | --- | --- | --- |
|  | r | P | r | P |
| Body weight | 0.476 | 0.340 | 0.394 | 0.382 |
| Lee index | 0.515 | 0.296 | -0.026 | 0.961 |
| Fat mass/Body weight (%) | -0.068 | 0.898 | 0.154 | 0.771 |
| Triglyceride (mmol/L) | 0.088 | 0.869 | 0.736 | 0.037^*^ |
| Total cholesterol (mmol/L) | 0.354 | 0.492 | 0.423 | 0.296 |
| High density lipoprotein (mmol/L) | -0.023 | 0.966 | -0.715 | 0.046^*^ |
| Low density lipoprotein (mmol/L) | 0.521 | 0.289 | 0.378 | 0.356 |
| Serum insulin | -0.774 | 0.071 | 0.879 | 0.004^**^ |
| Fasting blood glucose | -0.382 | 0.455 | 0.901 | 0.002^**^ |
| Glucose uptake | 0.189 | 0.719 | 0.777 | 0.023^*^ |
| HOMA-IRI | -0.779 | 0.068 | 0.912 | 0.002^**^ |

HOMA-IRI: homeostasis model assessment insulin resistance index. r = correlation coefficient (n = 8) ^*^P< 0.05, ^**^P<0.01
